# Goats on the Move: Evaluating Machine Learning Models for Goat Activity Analysis Using Accelerometer Data

**DOI:** 10.3390/ani14131977

**Published:** 2024-07-04

**Authors:** Arthur Hollevoet, Timo De Waele, Daniel Peralta, Frank Tuyttens, Eli De Poorter, Adnan Shahid

**Affiliations:** 1 IDLab, Department of Information Technology, Ghent University-imec, Technologiepark-Zwijnaarde 126, B-9052 Ghent, Belgium; arthur.hollevoet@ugent.be (A.H.); daniel.peralta@ugent.be (D.P.); eli.depoorter@ugent.be (E.D.P.); adnan.shahid@ugent.be (A.S.); 2 Faculty of Veterinary Medicine, Ghent University, D8, Heidestraat 19, B-9820 Merelbeke, Belgium; frank.tuyttens@ugent.be; 3 Flanders Research Institute for Agriculture, Fisheries and Food (ILVO), Burgemeester Van Gansberghelaan 92, B-9820 Merelbeke, Belgium

**Keywords:** accelerometer, animal monitoring, behavior classification, convolutional neural network, goats, machine learning, time series

## Abstract

**Simple Summary:**

This study developed and investigated a method to monitor animal activity by placing sensors on goats to better understand their behavior and improve their living conditions. Previous methods struggled to accurately classify complex movement data, but recent advancements in deep learning have made this task more effective. We compared the performance of three machine learning models: a basic neural network, a Convolutional Neural Network, and a hybrid model combining both. We evaluated two preprocessing methods to make the sensor data invariant to rotations, which could occur due to different placements of the sensors or slipping of the collars. Our results showed that the hybrid model, especially with these rotation invariant transformations, was the most accurate in identifying different goat activities. This research can help farmers monitor the health and well-being of their goats more effectively, ensuring better living conditions and improving dairy production. Our findings highlight the potential of advanced data analysis to enhance animal welfare and farm management practices.

**Abstract:**

Putting sensors on the bodies of animals to automate animal activity recognition and gain insight into their behaviors can help improve their living conditions. Although previous hard-coded algorithms failed to classify complex time series obtained from accelerometer data, recent advances in deep learning have improved the task of animal activity recognition for the better. However, a comparative analysis of the generalizing capabilities of various models in combination with different input types has yet to be addressed. This study experimented with two techniques for transforming the segmented accelerometer data to make them more orientation-independent. The methods included calculating the magnitude of the three-axis accelerometer vector and calculating the Discrete Fourier Transform for both sets of three-axis data as the vector magnitude. Three different deep learning models were trained on this data: a Multilayer Perceptron, a Convolutional Neural Network, and an ensemble merging both called a hybrid Convolutional Neural Network. Besides mixed cross-validation, every model and input type combination was assessed on a goat-wise leave-one-out cross-validation set to evaluate its generalizing capability. Using orientation-independent data transformations gave promising results. A hybrid Convolutional Neural Network with $L^{2}$-norm as the input combined the higher classification accuracy of a Convolutional Neural Network with the lower standard deviation of a Multilayer Perceptron. Most of the misclassifications occurred for behaviors that display similar accelerometer traces and minority classes, which could be improved in future work by assembling larger and more balanced datasets.

## 1. Introduction

In 2019, Europe held 1.9% of the world’s total goat population, but its total share of the worldwide goat milk production amounted to 15.1% [1]. This suggests that, in Europe, a limited population of goats is responsible for producing a significant share of goat dairy products. Consequently, goat farming plays a crucial role in the economy, society, and environmental sustainability. Moreover, the demand for goat milk is increasing, attributed to its sensory appeal and reduced allergenic properties. Additionally, dairy goat agriculture aligns with the United Nations 2030 Agenda for Sustainable Development, offering a pathway to meet global sustainability goals [2,3].

As the importance and the extent of goat farming will only increase in the near future, improving the living conditions of these farmed animals is essential, not only for the animals’ welfare but for the milk quality and production as well [4]. When aiming at improving animal welfare, monitoring farm animal behavior becomes a fundamental aspect. There are several ways to monitor animals, such as video recordings and periodically examining blood or milk samples [5]. However, these methods are labor-intensive, time-consuming, and need supervision, so they cannot be fully automated. For this reason, extensive research has been performed on animal activity recognition (AAR), which emerged from human activity recognition (HAR) [6]. AAR uses sensor data to provide insight into animals’ behavior (e.g., location, activities, and interactions) and environment (e.g., temperature, humidity, and light intensity) [7]. In this paper, we focused on AAR using sensor data from inertial measurement units (IMUs) attached to collars around the necks of goats. Linking IMU measurements to the correct activity with decent accuracy has been achieved using traditional classification methods such as Naive Bayes (NB) [8,9], k-Nearest Neighbours (kNN) [9,10], and Support Vector Machines (SVMs) [10,11]. These previous studies used small datasets with a low sampling rate and a limited number of classes, meaning the developed methods were not applicable to more complex time series datasets. While IMUs are effective for basic activity recognition, they have limitations in detecting more nuanced behaviors such as differentiating between standing and lying. Such distinctions are crucial for assessing conditions like pain or malaise, which are significant for animal welfare but may not be clearly reflected through simple accelerometer data.

Deep learning added several new possibilities to the field of AAR in comparison to traditional classification methods which are based solely on manually extracted time series features [12]. Table 1 lists an overview of the related research on both AAR and HAR, using traditional machine learning and deep learning methods. Although Multilayer Perceptrons (MLPs) and Convolutional Neural Networks (CNNs) have been well studied, a comparative analysis including both methods on the same dataset is, as far as what is currently known to the authors, non-existent. While an MLP often makes use of pre-extracted features, a CNN applies automated feature learning to self-extract relevant features from the data itself [13,14,15]. Subsequently, combining these models into a stacked generalization ensemble [16], or hybrid Convolutional Neural Network (hCNN) could improve performance even more. While not much research on hCNNs has been performed, the most important finding was an increase in terms of the balanced accuracy score when concatenating the self-learned features from the CNN with the manually extracted features when evaluated on different datasets [12,16,17].

Concerning the validation of the trained models, most of the proposed methods used a mixed cross-validation scheme, where the data from all individuals were merged and randomly split into training folds. Although this approach is useful for evaluating the performance of a method when faced with new data from the same individuals, it does not evaluate its generalization capabilities when confronted with new individuals, in which case, the performance tends to be severely overestimated. Therefore, some studies have applied leave-one-animal-out cross-validation (LOOCV) using the data from one individual for validation and the rest for training to better estimate the error when performing AAR on new individuals.

The decline in performance of such a generalized model can be attributed to two main factors: the reduction in the size of the training dataset and the variability in behavioral characteristics among different animals of the same species [13,14,18,21,23,24]. To solve this, orientation-independent methods like the $L^{2}$-norm, to calculate the vector magnitude, and the Discrete Fourier Transform (DFT) were introduced [25]. Although the first six DFT components have already been used as features in machine learning classifiers [16,21], entering all extracted components from a segment into a CNN has not been evaluated before for AAR.

This paper focused on gaining deeper insights into the learning behaviors of several machine learning-based classifiers in the field of AAR using IMU data captured from goats, using a publicly available dataset. We compare the results of a state-of-the-art CNN for AAR with an MLP, as well as with an hCNN that combines the CNN with the MLP. As a baseline, we will also show the results of classical machine learning algorithms (SVM and RF). For deciding which features to extract for the MLP, the hCNN, and the classical classifiers, we employed an automated feature selection strategy using the Kendall rank correlation coefficient [24]. Using an automated strategy removed the need for domain expertise, enabling a more streamlined and effective analysis. To gain better insights into the generalization capabilities of the different classifiers, we evaluated using both mixed cross-validation and animal-wise LOOCV. Furthermore, we looked into the effect the $L^{2}$-norm and the DFT transformation has on reducing the intra-species variability. Finally, due to the imbalanced nature of most AAR datasets, we focused on the balanced accuracy score when comparing the different strategies to each other. This is different from many papers focusing on classification tasks, as they often only report the overall accuracy, which could result in misleading conclusions when the datasets are imbalanced, as is often the case in AAR.

The main contributions of this paper are as follows:

- We combine an automated feature selection strategy with several machine learning algorithms to analyze goat activity data to extract time series features without any required domain expertise.
- We combine both time series data with scalar features in a hybrid CNN architecture to improve the performance of only using either the time series data or the scalar features.
- We provide and compare deeper insights into the learning behavior and generalization capabilities of several machine learning-based strategies for AAR using mixed cross-validation as well as animal-wise LOOCV.
- We evaluate the impact of calculating the $L^{2}$-norm and the DFT on the generalization capabilities of the different approaches.

The remainder of this paper is structured as follows. In Section 2, we will provide an overview of the used experimental approach, the used data, and the architecture of the machine learning models. Section 3 will go through the results for both the mixed and the goat-wise cross-validation methods, including an extensive discussion of these results. Finally, Section 5 will conclude the paper and provide some remarks on potential future work.

## 2. Methodology

**Figure 1:**
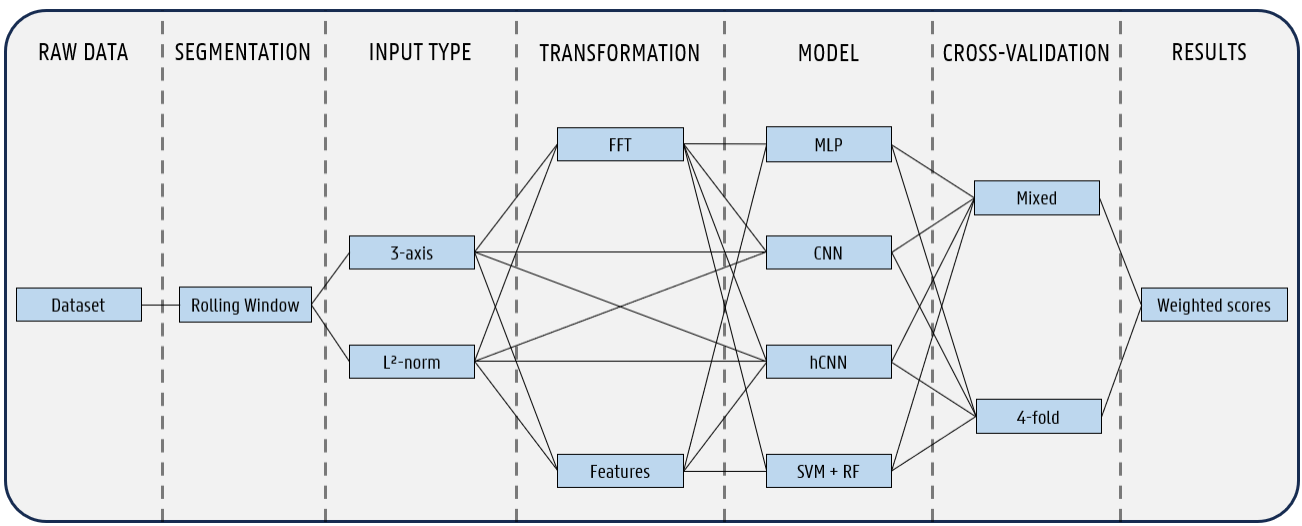
Overview of experimental approach.

First, we will give an overview of the data used, as well as discuss the steps taken to prepare the dataset for classification. We will then give an in-depth description of the several preprocessing steps taken to further process the dataset to make it well suited for the machine learning algorithms. Finally, we will conclude this section with an in-depth overview of these algorithms and models. A graphical overview of our experimental approach, including both the preprocessing and the evaluation, is visualized in Figure 1.

### 2.1. Dataset Description

**Figure 2:**
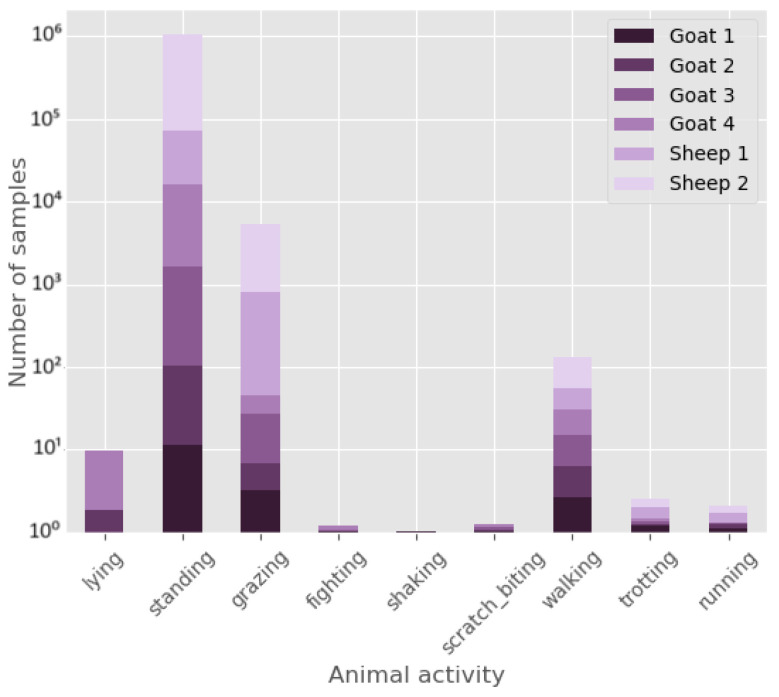
Sample distribution of original dataset across all animals per label.

For this study, an open-access dataset from Kamminga et al. [21] was used, consisting of IMU sensor data from four goats and two sheep. Goats and sheep are different from each other in size, weight, and other characteristics, but they belong to the same subfamily of Caprinae. The following description summarises the most relevant points of the paper written by Kamminga et al. to describe this dataset. The IMU sensors (Inertia ProMove-mini nodes [26]) were attached to a collar and placed in various rotations around the neck of each animal, making them susceptible to rotation and shifting. The sensors were synchronized in time with each other and were sampled at 200 Hz. The data were labeled using an application based on a Matlab GUI with video recordings used as a reference. The dataset contains nine activities: lying, standing, grazing, fighting, shaking, scratching–biting, walking, trotting, and running. Figure 2 displays the data distribution of samples across all six animals per label.

Next, we cleaned the dataset by maintaining only 5 out of the 18 columns from the dataset, removing the data related to the gyroscope and magnetometer. The remaining columns were the label, the timestamp, and the acceleration on the x-, y-, and z-axis. We opted to focus only on the accelerometer data to reduce the computational complexity, as earlier research in the field of AAR has already shown that just the accelerometer data were sufficient for the accurate classification of animal activities [12]. In total, the whole dataset consisted of 13,778,153 samples, or just under 20 h, distributed across the six animals, as displayed in Figure 2. Due to the limited amount of data captured from the sheep, we focused on only the data from the four goats.

The activity shaking was the most underrepresented in this dataset. It consisted of just 16,309 samples, or 80 s, while the next least represented activity, fighting, consisted of 75,209 samples, or just over 6 min. Another noteworthy aspect of the captured activities was the almost unnoticeable, from a signal processing perspective,  difference between the activities lying and standing. For both activities, the animals were stationary, resulting in a flat accelerometer signal. As a result of these issues, the activity shaking was entirely dropped from further evaluation, while lying and standing were combined into a single class called stationary. While the difference between standing and lying is often crucial to correctly assess the health and welfare of an animal, distinguishing between them was not feasible with only accelerometer data. By classifying the transitions between different activities and postures or adding more data such as gyroscope data, this limitation could be overcome. However, this was not within the scope of this study and should be looked at in future research.

### 2.2. Dataset Processing

#### 2.2.1. Segmentation

Previous research has shown that downsampling the data under 10 Hz decreases the accuracy significantly, but the effect of larger sampling rates (above 50 Hz) depends on the dataset itself [12,14,22,23,27]. Therefore, the original 200 Hz sampling rate of the dataset was maintained without downsampling. It has also been shown that increasing the length of the windows used for classification has a positive effect on accuracy [10,16,23]. Although longer segments contain more information, using time intervals longer than 10 s is not advised due to having fewer segments to train when splitting the dataset into a training and validation set [18]. The cleaned dataset was split up into smaller segments of equal length, each corresponding to a single label by removing segments with two or more labels that represented transitions between different activities. The rolling window method was used using a step size of half the window length. We used a window size of 3 s, equal to 600 samples at a sampling rate of 200 Hz (N=600) after earlier studies had shown that these parameters performed best in terms of classification performance [10,16,23]. The resulting data distribution is shown in Table 2.

#### 2.2.2. Input Sample Description

The collars, to which the IMUs were attached, were prone to slipping. Therefore, two techniques were used to make the data more orientation-independent. The first method was calculating the magnitude of the acceleration vector through the $L^{2}$-norm, defined as

(1) $$M\left(t\right)=\sqrt{a_{x}{\left(t\right)}^{2}+a_{y}{\left(t\right)}^{2}+a_{z}{\left(t\right)}^{2}}$$

where $a_{x}$, $a_{y}$, and $a_{z}$ represent the individual sample of each respective axis of the accelerometer at time *t*.

The second method was transforming the data from the time domain to the frequency domain by calculating the DFT [25]. This was performed using the Fast Fourier Transform (FFT) function of the Scipy Python package, which calculates the 1D n-point DFT of a real input [28]. The algorithm is defined in Scipy as follows:

(2) $$X_{k}=\sum_{n=0}^{N-1}x_{n}\cdot e^{-\frac{i2\pi }{N}kn}$$

where *k* is the index of the frequency component and $X_{k}$ is the magnitude of that component. *x* is a 1D-array, or, in other words, the segment, with length *N* and *n* is the index of an element in *x*. The maximum number of frequency components that can be extracted from a purely real input is half of the number of samples in the segment due to the Hermitian symmetric output and Nyquist theorem [28].

After calculating both the vector magnitude and the DFT, we obtained 4 different versions of the accelerometer data: the raw 3-axis data and their $L^{2}$-norm variant, each in both the time domain and the frequency domain. The time-domain data were standardized (Z-score) independently for each goat to further remove some of the intra-animal variations, and the frequency domain data were normalized between 0 and 1.

#### 2.2.3. Feature Engineering

To evaluate the difference between applying machine learning on the raw time series versus training on extracted features, we applied an automated feature selection strategy as proposed by De Waele et al. [24]. To extract features from time series data, we used the *tsfel* Python package (https://www.python.org/) [29]. This library automatically extracts a wide variety of time series features. In total, 389 features per axis were calculated; Table 3 gives a broad overview of the types of features extracted.

Removing features that either generated bins, such as histograms, or that were constant throughout time, resulted in a remaining 53 features per axis. Next, to avoid the curse of dimensionality, we used the Kendall rank correlation coefficient which determines the statistical significance of each feature with respect to the classification labels [24]. This coefficient is based on the number of concordant and discordant sample pairs. From the correlation coefficient τ, the statistical *p*-value can be determined. A low *p*-value means a high probability of correlation between the feature and the labels. The algorithm was applied to the extracted $L^{2}$-norm features and the *p*-value threshold was set to p≤0.05. Out of the 53 extracted $L^{2}$-norm features, a total of 44 were deemed statistically relevant. These features were then also extracted for the x-, y-, and z-axis. The algorithm computing the Kendall Tau coefficient and its corresponding *p*-value is depicted in Algortihm 1. The final feature values were scaled by a RobustScaler from the sklearn Python library, which is normalization using the median and the interquartile range, and a power transformer based on the Yeo–Johnson method [30]. This was performed to ensure that all features followed similar distributions, making them better suited for handling by machine learning algorithms.
**Algorithm 1:** Algorithm used by [24] to compute the Kendall Tau coefficient and corresponding *p*-value.
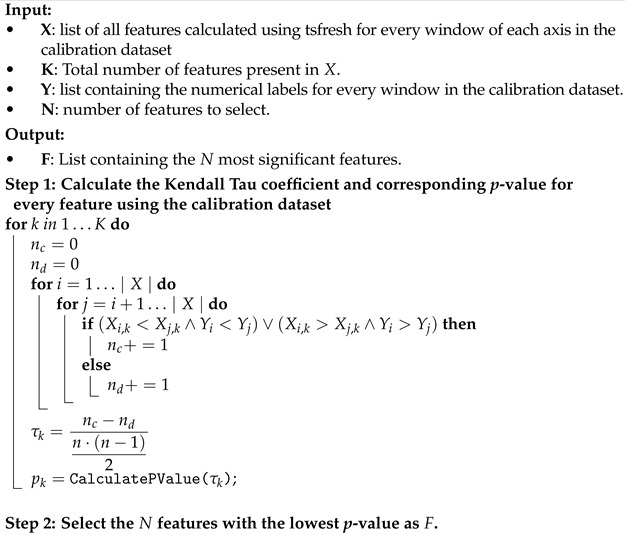


### 2.3. Machine Learning Models

#### 2.3.1. Convolutional Neural Network (CNN)

**Figure 3:**
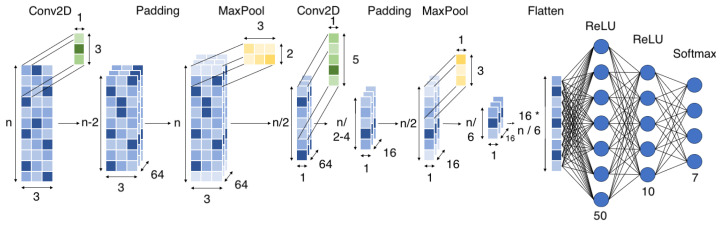
Graphical overview of the CNN architecture used in this study. For each layer we provide the dimensions of the input and kernels in all 3 dimensions. Here *n* represents the input sample size (600 for the time-domain and 300 for the frequency-domain. On top the layers applied are being shown.

Convolutional layers naturally carry out unsupervised feature learning by sliding a filter over the input and convolving the values. These generated features are subsequently transformed to either undergo another convolution or sent to a stack of fully connected layers for classification [16]. The CNN architecture is mainly based on the proposed architecture from two papers by Eerdekens et al. [12,23]. Both papers suggested a two-layered CNN because studies demonstrated adding more layers made the model extract more complex features, reducing the accuracy of the validation data due to overfitting [12,17]. Preliminary results from our experiments also showed a 2-layer CNN to be the best-performing architecture for goat activity classification. The architecture used in this study is depicted in Figure 3 with two convolutional layers, each followed by a max-pooling layer and a dropout layer. The learned features from the convolutional layers were then flattened and passed through two fully connected layers for classification. Table 4 gives an overview of all used hyperparameters.

Before training the model, the 7 activities used in this study were encoded as sparse labels ranging from 0 to 6. As the dataset contained highly imbalanced data, running the risk of the model being biased towards the majority class and performing worse when it comes to classifying the minority classes, we opted to apply a weighted loss function. This adds more weight to the terms in the loss function coming from the minority classes [12]. These weights were calculated as follows:

(3) $$w_{classes,k}=\frac{n_{instances}}{n_{classes}\cdot n_{instances,k}}$$

where $n_{instances}$ is the total number of samples, $n_{classes}$ is the number of classes, and $n_{instances,k}$ is the number of samples for a class *k*.

#### 2.3.2. Multilayer Perceptron (MLP)

**Figure 4:**
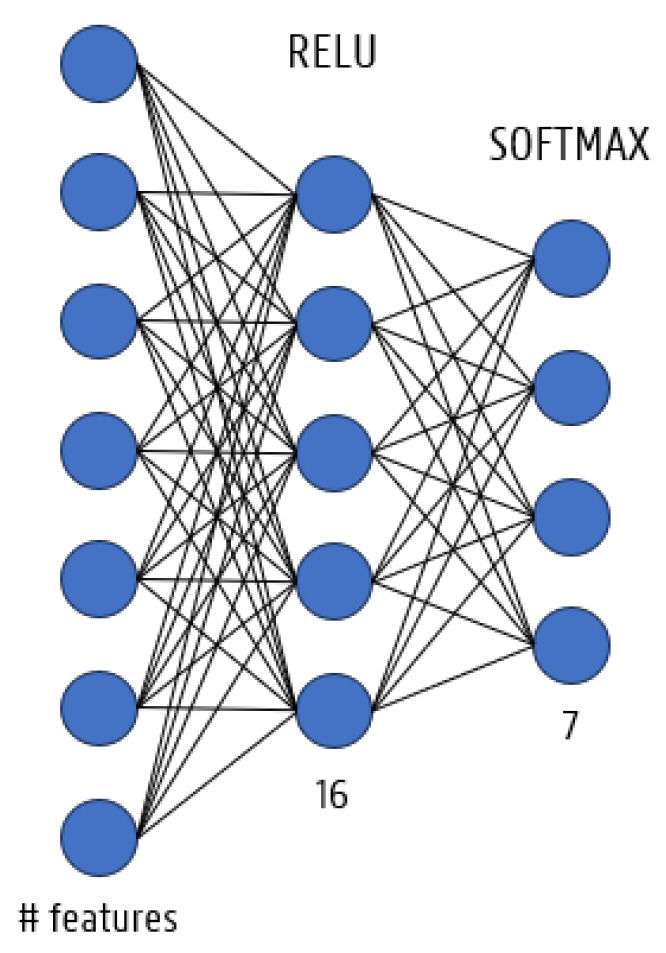
The used MLP architecture.

An MLP is an artificial neural network consisting of an input layer, one or more hidden 245 layers (=fully connected layers), and an output layer enabling the network to pick up 246 complicated, non-linear connections between inputs and outputs. The first layer has as many neurons as there are features while the single hidden layer has a fixed number of neurons of 16, as depicted in Figure 4. The other learning hyperparameters were the same as the CNN.

#### 2.3.3. Hybrid Convolutional Neural Network (hCNN)

An hCNN is essentially a deep learning ensemble model that combines a CNN and MLP at the fully connected layer level. Both the CNN and MLP used in this model are the same as in previous sections. A CNN captures local patterns, whereas the MLP searches for patterns in the global characteristics of the segment, so combining their advantages into an ensemble could improve classification accuracy [12,16,17]. The outputs of both the CNN and the MLP were then concatenated, resulting in a total size of 26 neurons (10 + 16). Finally, a classification layer with the Softmax activation function was added to obtain the final output of the model. The training hyperparameters were the same as those for the CNN and the MLP.

#### 2.3.4. Baseline Classifiers

To ensure the proposed deep learning models generalize better than traditional classifiers, their results were compared against an SVM and RF. The two main hyperparameters of an SVM are the regularization parameter C and the used kernel function. Both were kept at the standard values of 1.0 for C, and *rbf* as the used kernel. For the RF, the number of trees and the criterion were kept as default, 100 and the Gini criterion, respectively. For the number of features considered at each split, we opted for using all features instead of the square root of the number of features after the preliminary results showed this to be the better option in terms of performance.

## 3. Results

All models were trained and evaluated using a stratified 8-fold cross-validation, referred to as mixed cross-validation, to investigate how the models generalize towards data coming from the same animals as in the training dataset. We also evaluated them with a goat-wise leave-one-out cross-validation to assess the generalization capabilities to new, unseen during training, animals of the proposed models. To correctly assess the performance of each of the evaluated models, careful consideration should be given to the evaluation criteria that were used. In this study, balanced accuracy in combination with confusion matrices was used to assess the model performance. We noticed that most studies used unbalanced accuracy but in the case of heavily imbalanced datasets, like is often the case in AAR, this metric can make the model performance look better than it is. This can be the case if the majority class has a much higher prediction accuracy than the minority classes. The balanced accuracy is calculated as follows:

(4) $$ACC_{balanced}=\frac{\sum_{k=0}^{N_{c}}\frac{TP_{k}}{TP_{k}+FN_{k}}}{N_{c}}$$

where $N_{c}$ is the total number of classes, *k* is the respective class, and $TP_{k}$ and $FN_{k}$ stand for the number of true positive and false negative predictions of that class. This is the same as taking the average over all the classes of the recall per class. Confusion matrices were also used to evaluate each model and to spot the classes that are difficult to classify.

### 3.1. Mixed Cross-Validation

Table 5 gives an overview of the results from the mixed cross-validation evaluation. The first thing we observed was the three-axis input being the better input overall. In this case, the best-performing algorithms for the time and frequency domain, the MLP (94.4%±1.2) and the hCNN (94.3%±1.1), respectively, were both within each other’s range and boasting accuracies of well above 90%. While the CNN-based models did not show a significant difference in performance between the time and frequency domains, this was not the case for the MLP and the classical machine learning algorithms. This may suggest a potential misalignment between the transformed features and the classifiers’ learning mechanisms.

When converting the input data from the three-axis input type to the $L^{2}$-norm, similar conclusions could be made, albeit the performance metrics report some percentage points lower than for the three-axis input data. This could indicate that too much information was lost when reducing the dimensionality of the data. In this case, the difference between the time and frequency domain also became more outspoken. This was potentially due to a further misalignment between the data and the learning mechanisms of the classifiers, as the gap between the time and frequency domain was the largest for the classifiers that were the worst performing in the frequency domain for the three-axis data (MLP, SVM, and RF).

### 3.2. Goat-Wise Cross-Validation

When examining Table 6, more insightful observations about the generalization capabilities of the models could be made. As expected, the balanced accuracy decreased while standard deviations increased in comparison to their respective values for mixed cross-validation. The reason was that in mixed cross-validation, all goats were represented in the training data, while goat-wise cross-validation validated the model on a new goat. Each goat likely had a unique movement style and attachment orientation for the IMU and thus the data distribution for this goat slightly varied from its fellow goats. When using mixed validation, the models have already seen data from each goat and thus have already used all available distributions to optimize its parameters. Due to the increase in variability of the results over multiple iterations and the fold, forming a conclusive statement about the generalization capability of the models and input types was less ambiguous.

One of the first things we noticed, for the three-axis input data, was that the frequency domain outperformed the time domain data in most cases. Not only did it show higher balanced accuracy metrics but it also boasted lower standard deviations. This could be a sign that transforming the data from the time domain to the frequency domain through the DFT removed some of the intra-goat variability and thus made the models generalize better to unseen animals. This hypothesis was further strengthened when we looked at the $L^{2}$-norm data, which further improved the performance and reduced the standard deviation for most models, except for the CNN and the SVM. These two findings strengthen our hypothesis that the $L^{2}$-norm and DFT are necessary preprocessing methods to make the IMU data less rotation-dependent and make the models generalize better.

When using the goat-wise LOOCV on the $L^{2}$-norm, both the hCNN and CNN outperformed most of the other algorithms, especially with the data in the frequency domain. Only the MLP using time domain features could match the performance of the CNN-based models. While there was a significant performance improvement when using the hCNN over the CNN using mixed cross-validation, this difference was mostly gone when using goat-wise LOOCV.

The large difference in performance between the mixed cross-validation and goat-wise LOOCV was a clear sign that special care should be taken when devising validation strategies for machine learning models. This is especially the case when working with AAR or healthcare data, as data will come from multiple individuals who all have slightly different characteristics, and the developed algorithm should generalize toward unseen subjects.

**Figure 5:**
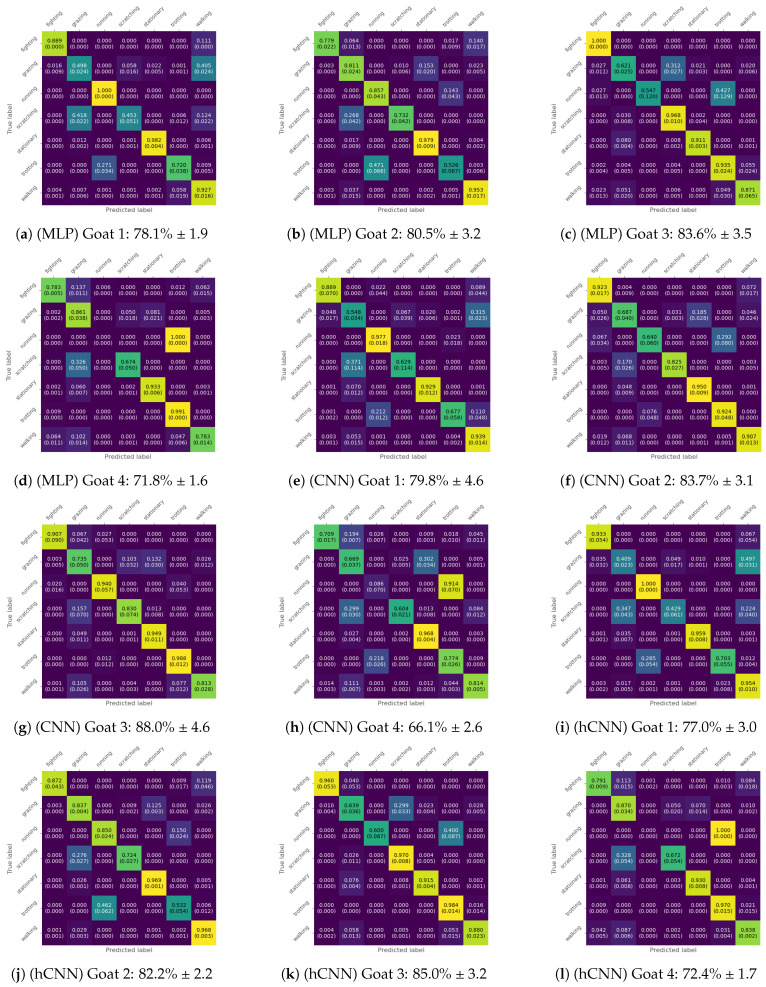
Confusion matrices showing the results of the goat-wise LOOCV.

Taking a closer look at the confusion matrices, we can determine for which activities the models had the most difficulty predicting correctly. For the sake of simplicity, we decided to show the confusion matrices only for the best resulting input type for each model evaluated. The confusion matrix for the MLP in the time domain, and the CNN and hCNN in the frequency domain, all using the $L^{2}$-norm, are shown in Figure 5, respectively, a–d, e–h, and i–m. In the case of the hCNN, we opted for the frequency domain as it gave a slightly smaller standard deviation value whilst the mean value was within the margin of error.

First, evaluating the second and third goats seemed to result in the highest accuracy, with the first goat typically being within the same range. This could indicate that the data distribution for each activity from those three goats was similar. The fourth goat performed worse over all three configurations, partially due to the misclassification of *running* as *trotting*. This could be due to the rarity of the *running* class and the lack of more individual animals in the training dataset, which could provide a wider variety of examples for this type of movement to improve the generalization capabilities of the models. Second, there seemed to be confusion between the activities *running* and *trotting* and the activities *scratching* and *grazing* because these were displayed as similar traces on the IMU data as these only captured movements from the head and neck of the goats and not from the legs or other body parts. Next, *grazing* was often misclassified as other low-intensity activities such as *walking*, *scratching*, and *stationary*. A reason for this could be that *grazing* shares many characteristics with those behaviors since a goat could eat whilst walking or standing still. The head movements could also be confused with scratching, as these were also similar. Lastly, it must be mentioned that the dataset balancing method used, using class weights in the loss function, made the models not overfit towards either majority or minority class. Overfitting towards the majority class stationary could occur if the adjusted weights were not implemented and overfitting towards the minority class could happen if the adjusted weights were scaled too strongly.

## 4. Discussion

In this paper, we obtained a multitude of valuable insights that should be taken into consideration when applying machine learning methodologies to the field of AAR. From the results of the goat-wise LOOCV, we observed that both the $L^{2}$-norm data and the DFT transformed data improved the performance over the three-axis and the time domain data for most of the models that were evaluated. There could be multiple reasons for this, as follows:

- The $L^{2}$-norm data severely reduce data dimensionality. Applying this norm to the three-axis data essentially compresses the data, reducing the amount of noise present in the signal. This made it easier for the models to focus on the relevant parts of the signal for discerning between the different classes.
- The $L^{2}$-norm made the data more rotation invariant, thus removing differences in the data distribution between the animals that may have occurred due to the placement of the neck collar. It also removed any shift in the data distribution that may have occurred in the signal due to the collar slipping over time.
- By applying the DFT, we removed the variation that could be present in the signal due to small time shifts, as most movements are periodic. This makes it easier for the models to find structures in the data and thus makes it easier to learn from the data and generalize as well.

These insights regarding the increased generalization capabilities when using the right preprocessing methods also have substantial implications for enhancing goat welfare and monitoring. The improved accuracy in identifying goat activities through these preprocessing methods allows for the earlier detection of health issues due to changes in their typical behaviors. Such advancements can facilitate continuous and automated welfare assessments, especially in extensive farming scenarios, thereby ensuring any signs of distress or illness are promptly addressed. Furthermore, these insights pave the way for the development of adaptive management practices and evidence-based welfare standards, optimizing care and enhancing the well-being of goats. By integrating these machine learning methodologies into everyday monitoring and management practices, we can achieve a deeper understanding of goat behavior, leading to more informed and welfare-oriented decision-making. To further validate the robustness and applicability of our models, future studies should focus on deploying these systems in diverse, real-world farm environments, allowing us to assess their performance under various uncontrolled conditions and adapt the models to effectively handle real-world variability.

## 5. Conclusions

In conclusion, this study has shed light on the intricacies of designing and evaluating machine learning models for AAR, with a specific focus on goat behavior. Our research demonstrated that models such as the hybrid CNN, along with specific data transformations, such as the $L^{2}$-norm and DFT, significantly improve the ability to generalize across different situations by minimizing errors caused by sensor movement and general noise. Furthermore, our findings emphasized the crucial role of the dataset-splitting methodology in model evaluation. We have highlighted the deficiencies of mixed validation and presented the merits of goat-wise LOOCV, which offers more reliable performance metrics. This work serves as a guidepost for future research in AAR, advocating for the careful consideration of data preprocessing and validation techniques to ensure the development of robust machine learning applications in this domain.

Future work in this field should aim to explore larger, more diverse datasets to validate the scalability and robustness of the findings presented. There is also a promising avenue to investigate the interpretability of machine learning models for AAR, understanding how these models make decisions, and ensuring their reliability in varied scenarios. Additionally, the integration of different sensor types and locations may offer a more comprehensive view of animal behavior, which could lead to the development of more nuanced and precise AAR systems. It would also be beneficial to assess the impact of real-time data processing and the feasibility of deploying these models in on-farm conditions to enhance their practicality and efficacy in real-world applications.

## Figures and Tables

**Table 1:** Overview of related research.

Study	Species	Classifiers ^a^	Features
[12]	Horse	RF, CNN, hCNN	Manually selected (3-axis, multiple sensors), ranked (RF classifier)
[18]	Goat	DT, NB, (SVM, MLP, LDA, kNN)	Manually selected (*L*^2^-norm), ranked (Chi-squared test)
[16]	Human	CNN, hCNN	No features (3-axis)
[19]	Sheep	LDA, QDA	Manually selected (3-axis), ranked (greedy feature selection)
[20]	Cow	DT, LDA, NB, kNN	Manually selected (3-axis)
[21]	Goat, sheep	DT, MLP, SVM, NB, LDA, kNN	Manually selected (*L*^2^-norm), ranked (Relief Algorithm)
[22]	Cow	NB, SVM, kNN	Manually selected (3-axis)
[23]	Horse	CNN	No features (3-axis)
[13]	Goat, horse	CNN	No features (3-axis, multiple sensors)
[14]	Horse	CNN	No features (3-axis, multiple sensors)
[17]	Sheep	CNN, hCNN	Manually selected (3-axis, *L*^2^-norm)
[24]	Horse	Clustering	ranked (Kendall Tau algorithm)
Our work	Goat	MLP, CNN, hCNN, (RF, SVM)	Combination of no features (3-axis, *L*^2^-norm) and features (3-axis,
			*L*^2^-norm, FFT components), Ranked (Kendall Tau algorithm)

^a^ DT: Decision Tree, NB: Naive Bayes, LDA: Linear Discriminant Analysis, QDA: Quadratic discriminant analysis, RF: Random Forest.

**Table 2:** Segment distribution per activity across all goats. Each segment contains 600 accelerometer samples.

Activity	Goat 1	Goat 2	Goat 3	Goat 4	Total
Stationary	3505	4056	3968	5711	17,240
Grazing	1673	1125	1948	787	5533
Walking	1351	1244	1265	1032	4892
Trotting	260	68	99	114	541
Running	157	169	30	7	363
Scratching	34	79	93	95	301
Fighting	9	47	15	164	235
Total	6989	6788	7418	7910	29,105

**Table 3:** Categories of features extracted by the tsfel Python library.

Statistical	Mean, median, standard deviation, variance, …
Temporal	Autocorrelation, energy, zero-crossing rate, …
Spectral	Spectral entropy, spectral centroid, spectral roll-off, …
Wavelet	Wavelet energy, wavelet entropy, …

**Table 4:** Hyperparameters used for training the CNN.

Parameter	Value
Size of input vector (time domain)	600 (3 s at 200 Hz)
Size of input vector (frequency domain)	300
Maximum epochs	400
Batch size	128
Number of feature maps	64–16
Filter size	3×1–5×1
Vertical stride	1–1
Horizontal stride	1–1
Max-pooling size	2×3–3×1
Dense layer size	5–10–7
Activation function	ReLU and Softmax (last layer)
Weight decay	0.01 (L2 regularisation)
Dropout rate	0.6
Optimizer	Adam

**Table 5:** Overview of the results for mixed cross-validation. Expressed as balanced accuracy and standard deviation.

Breakdown		Average Balanced Accuracy and Standard Deviation (%)				
Input Type	Domain Type	hCNN	CNN	MLP	SVM	RF
3-axis	Time	93.5 ± 1.3	88.7 ± 1.7	94.4 ± 1.2	92.6 ± 1.8	89.9 ± 2.4
	Frequency	94.3 ± 1.1	89.8 ± 1.2	83.2 ± 2.0	80.8 ± 1.7	79.0 ± 1.5
$L^{2}$-norm	Time	88.4 ± 1.5	81.2 ± 2.0	90.1 ± 1.9	88.5 ± 1.5	86.9 ± 1.8
	Frequency	91.4 ± 1.7	87.8 ± 1.9	75.9 ± 1.8	70.0 ± 2.0	79.4 ± 1.4

**Table 6:** Overview of the results for goat-wise leave-one-out cross-validation. Expressed as balanced accuracy and standard deviation.

Breakdown		Average Balanced Accuracy and Standard Deviation (%)				
Input Type	Domain Type	hCNN	CNN	MLP	SVM	RF
3-axis	Time	61.9 ± 22.3	35.6 ± 16.4	66.7 ± 14.7	61.8 ± 6.6	68.2 ± 3.5
	Frequency	70.2 ± 10.8	77.1 ± 6.6	65.7 ± 6.5	67.8 ± 8.6	65.0 ± 9.0
$L^{2}$-norm	Time	79.3 ± 5.6	70.2 ± 8.6	78.0 ± 4.9	65.0 ± 8.5	68.7 ± 9.5
	Frequency	79.1 ± 5.0	79.4 ± 8.4	66.2 ± 5.2	59.9 ± 5.9	68.9 ± 8.8

## Data Availability

The dataset used in this study was an open-source dataset called Multi Sensor-Orientation Movement Data of Goats. It can be accessed through the following URL: https://lifesciences.datastations.nl/dataset.xhtml?persistentId=doi:10.17026/dans-xhn-bsfb (accessed on 24 June 2024).
